# Soil Properties Correlate with Microbial Community Structure in Qatari Arid Soils

**DOI:** 10.1128/spectrum.03462-22

**Published:** 2023-02-27

**Authors:** Sini Skariah, Sara Abdul-Majid, Anthony G. Hay, Anushree Acharya, Noora Kano, Raghad Khalid Al-Ishaq, Paul de Figueiredo, Arum Han, Adrian Guzman, Soha Roger Dargham, Saad Sameer, Gi Eun Kim, Sabiha Khan, Priyamvada Pillai, Ali A. Sultan

**Affiliations:** a Department of Microbiology and Immunology, Weill Cornell Medicine—Qatar, Cornell University, Qatar Foundation—Education City, Doha, Qatar; b Department of Microbiology, Cornell University, Ithaca, New York, USA; c Department of Microbial Pathogenesis and Immunology, College of Medicine, Texas A&M Health Science Center, Texas A&M University, Bryan, Texas, USA; d Department of Veterinary Pathobiology, Texas A&M University, College Station, Texas, USA; e Department of Electrical and Computer Engineering, Texas A&M University, Texas, USA; f Department of Biomedical Engineering, Texas A&M University, Texas, USA; g Biostatistics, Epidemiology, & Biomathematics Research Core, Weill Cornell Medicine—Qatar, Cornell University, Qatar Foundation—Education City, Doha, Qatar; Migal-Galilee Research Institute

**Keywords:** microbiota, 16S rRNA gene sequencing, soil, desert habitats, soil chemical composition

## Abstract

This is the first detailed characterization of the microbiota and chemistry of different arid habitats from the State of Qatar. Analysis of bacterial 16S rRNA gene sequences showed that in aggregate, the dominant microbial phyla were *Actinobacteria* (32.3%), *Proteobacteria* (24.8%), *Firmicutes* (20.7%), *Bacteroidetes* (6.3%), and *Chloroflexi* (3.6%), though individual soils varied widely in the relative abundances of these and other phyla. Alpha diversity measured using feature richness (operational taxonomic units [OTUs]), Shannon’s entropy, and Faith’s phylogenetic diversity (PD) varied significantly between habitats (*P* = 0.016, *P* = 0.016, and *P* = 0.015, respectively). Sand, clay, and silt were significantly correlated with microbial diversity. Highly significant negative correlations were also seen at the class level between both classes *Actinobacteria* and *Thermoleophilia* (phylum *Actinobacteria*) and total sodium (*R* = −0.82 and *P* = 0.001 and *R* = −0.86, *P* = 0.000, respectively) and slowly available sodium (*R* = −0.81 and *P* = 0.001 and *R* = −0.8 and *P* = 0.002, respectively). Additionally, class *Actinobacteria* also showed significant negative correlation with sodium/calcium ratio (*R* = −0.81 and *P* = 0.001). More work is needed to understand if there is a causal relationship between these soil chemical parameters and the relative abundances of these bacteria.

**IMPORTANCE** Soil microbes perform a multitude of essential biological functions, including organic matter decomposition, nutrient cycling, and soil structure preservation. Qatar is one of the most hostile and fragile arid environments on earth and is expected to face a disproportionate impact of climate change in the coming years. Thus, it is critical to establish a baseline understanding of microbial community composition and to assess how soil edaphic factors correlate with microbial community composition in this region. Although some previous studies have quantified culturable microbes in specific Qatari habitats, this approach has serious limitations, as in environmental samples, approximately only 0.5% of cells are culturable. Hence, this method vastly underestimates natural diversity within these habitats. Our study is the first to systematically characterize the chemistry and total microbiota associated with different habitats present in the State of Qatar.

## INTRODUCTION

Soil microbes perform essential biological functions, including organic matter decomposition and nutrient cycling, maintenance of energy equilibrium, soil structure preservation, water purification, plant nutrition, and disease suppression (1). In hot deserts, the collective effects of high temperatures and limited moisture availability lead to unique adaptations, allowing microbes to persist in these extreme niches (2, 3). The State of Qatar is a subtropical desert, on the western coast of the Arabian Gulf, characterized by arid and hot weather conditions with scanty rainfall (81 mm/year) (4, 5). It is considered a climate change hot spot and is predicted to experience increases in extreme events, including high heat (up to 8°C rise) and drought, in the coming years (6). Such temperature changes, coupled with strong urbanization pressure (7) and active expansion of agricultural activities in the State of Qatar (8), are likely to strain Qatari ecosystems.

Qatar, despite being a relatively small country, harbors diverse habitats that range from terrestrial dunes, sabkhas, micronebkhas, rodahs, and wadis to marine environments, including mangrove forests (4, 9, 10). Limestone and gypsum cover the majority of the country’s surface, and about 88% of the country is a flat or gently undulating rocky desert (4, 11–13). Sabkhas are flat, saline, and gypsiferous areas that are formed when intense water evaporation occurs from low-lying areas inundated with seawater or rainwater, leaving behind a dried salt-encrusted surface (12, 14, 15). Approximately 6% of the land in Qatar is covered by sabkhas that occur mostly within coastal regions but are also sporadically found inland (14–16). They are composed of silt, clay, muddy sand, and evaporate minerals like gypsum, anhydrite, and halite (14, 17). Micronebkhas are phytogenic hillocks that rarely exceed 50 cm in height and are seen on the margins of some sabkhas (17, 18). Rodahs, meaning “gardens” in Arabic, are fertile depressions containing nutrient-rich soil that often support dense vegetation compared to surrounding elevations (15). Depression soils like these constitute about 2.4% of Qatari soils (4, 15). Depressions in the northern region of the country are more numerous, irregular/linear in shape, and fine textured, whereas the southern depressions are small and circular, with coarser aeolian sand deposits (4, 12, 15). Wadis, meaning “valleys” in Arabic, are watercourses that are flash flooded during the rainy seasons (19). Barchan sand dunes are mobile, moving from tens to hundreds of meters a year depending on their size, and dominate the southeastern region of the State of Qatar. Arable land, however, constitutes only 1.2% of Qatar’s total land area, with most of the approximately 800 local farms usually set up on rodahs where groundwater is available (20, 21).

Qatari soils generally have an alkaline pH (7.6 to 9.8) and low moisture content (0.2 to 8.8%) (4, 10, 22). Higher water holding capacity and organic carbon percentage are usually seen in rodahs and wadis than in sabkhas and sand dunes (4). In salt marshes, the inundated areas, like mangroves and lower marshes, feature higher electrical conductivity (EC), soil moisture, total carbonate, and sodium levels and lower pH (23). Cultivated soils mostly have higher organic matter content and nutrients than other desert soils and often support non-native and naturalized vegetation species (12). Abiotic factors, including sodium, lead, potassium, and magnesium, were previously shown to correlate with microbial composition variance in Qatari sand dunes (24), but little is known about the correlations between chemical and microbial community composition elsewhere in the state.

A number of studies focusing on cultured microbes from specific habitats in Qatar have been published previously. Al-Thani and Yasseen found that the most abundant cultured bacterial taxa in arid Qatari soils were *Actinobacteria*, *Firmicutes*, and *Proteobacteria* (10). Mangrove forest sediments in Qatar are dominated by cellulase-producing bacteria that are influenced by water temperatures (10, 25–27), while *Streptomyces* are common in Qatari arid habitats, including rodahs (30 to 40% of total CFU) (22). Sabkhas contain an abundance of cultured extremophiles such as halothermophilic bacteria and heterocyst *Cyanobacteria* (28, 29). Previously, we reported on bacterial communities in Qatari dunes studied through direct counts, cultivation, and 16S rRNA gene and metagenomic sequence analyses. We found that our cultured isolates were either *Actinobacteria* (58%), *Firmicutes* (27%), or *Proteobacteria* (15%), with culturability ranging from 0.4 to 3.8% depending on the methodology used (24). Deep sequencing of 16S rRNA gene amplicons revealed that proteobacterial DNA, particularly from enteric bacteria, was (on average) most abundant but showed dune-specific patterns that correlated with dune size and proximity to camel farms (24).

Although several studies have quantified culturable microbes in selected Qatari habitats and worldwide, the approach has serious limitations (22, 23, 25, 26). In environmental samples, approximately only 0.5% of cells are culturable (22–24, 28–33); hence, this method vastly underestimates natural diversity within these habitats. Given that Qatar is one of the most hostile and fragile environments on Earth (5, 9) and is expected to face a disproportionate impact of climate change in the coming years, it is critical to establish a baseline understanding of microbial community composition across different habitats and to assess how soil edaphic factors (texture and chemical properties) correlate with microbial community composition. This understanding may help us to estimate future ecosystem responses to environmental changes and provide clues on how they may impact human life. This study is the first to systematically characterize the soil properties and total microbiota associated with different habitats present in the State of Qatar.

## RESULTS

**FIG 1:**
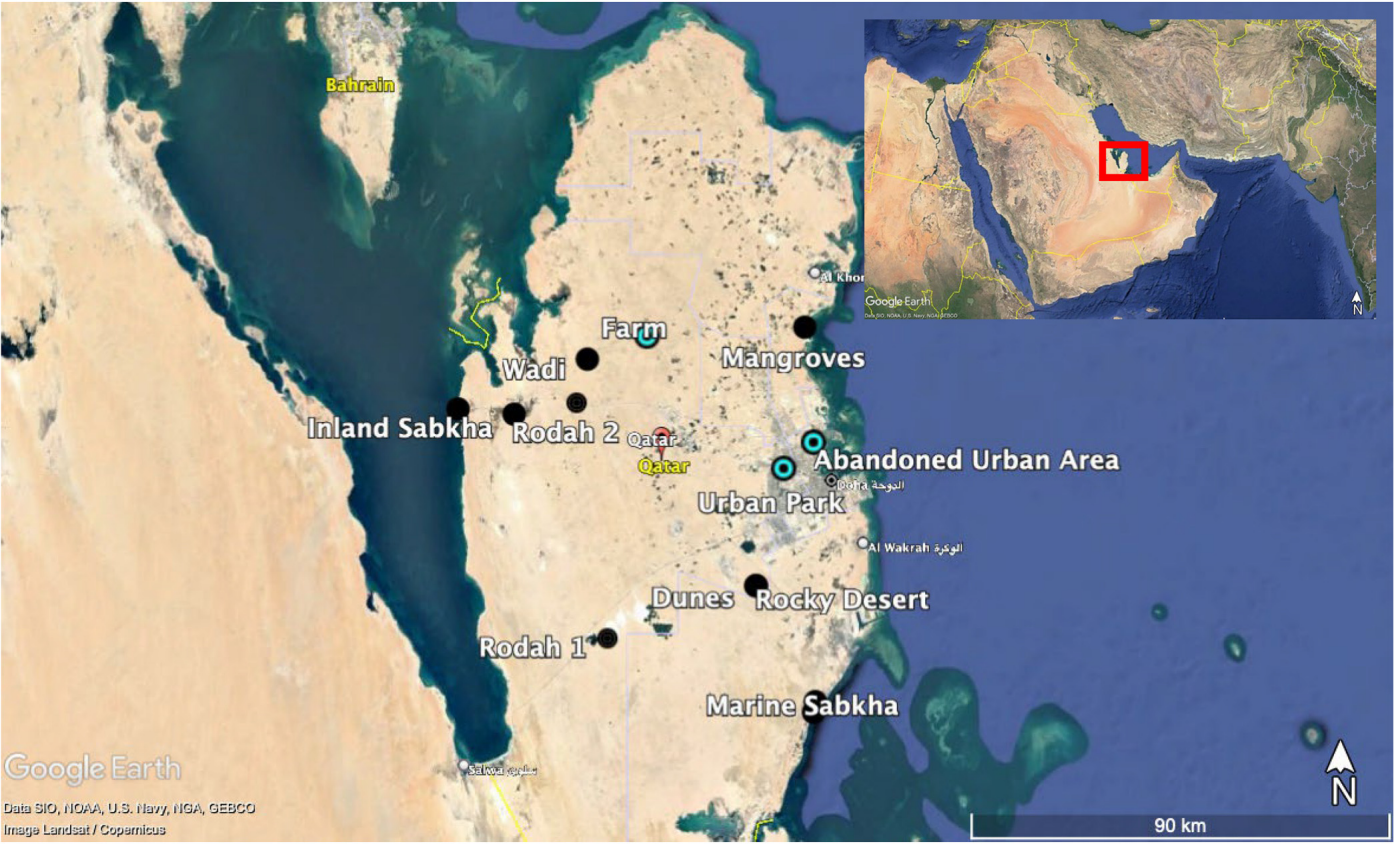
Google Earth image showing soil sample collection location points from various natural (black circles) and human-impacted (cyan/black bullseyes) habitats around the State of Qatar. The red box in the inset shows the geographic location of Qatar.

A total of 26 soil samples from 13 habitats (10 natural and 3 human impacted) were collected in an effort to capture the different types of habitats present in the arid State of Qatar (Fig. 1). This included barchan sand dune, rocky desert, marine and inland sabkhas, two types of rodah, mangroves, wadi, micronebkhas, marine sand, farm, urban park, and abandoned urban area (see description in Table S1 in the supplemental material). Soil and air temperatures at the collection sites ranged from 26 to 49°C and 28 to 42°C, respectively, with the highest temperatures being recorded for inland sabkha and the lowest at the mangrove habitat (Table S1). While the majority of the sampled habitats were vegetated, the barchan dune, marine sand, and inland and marine sabkha habitats were not (Table S1).

### Microbial community composition.

**FIG 2:**
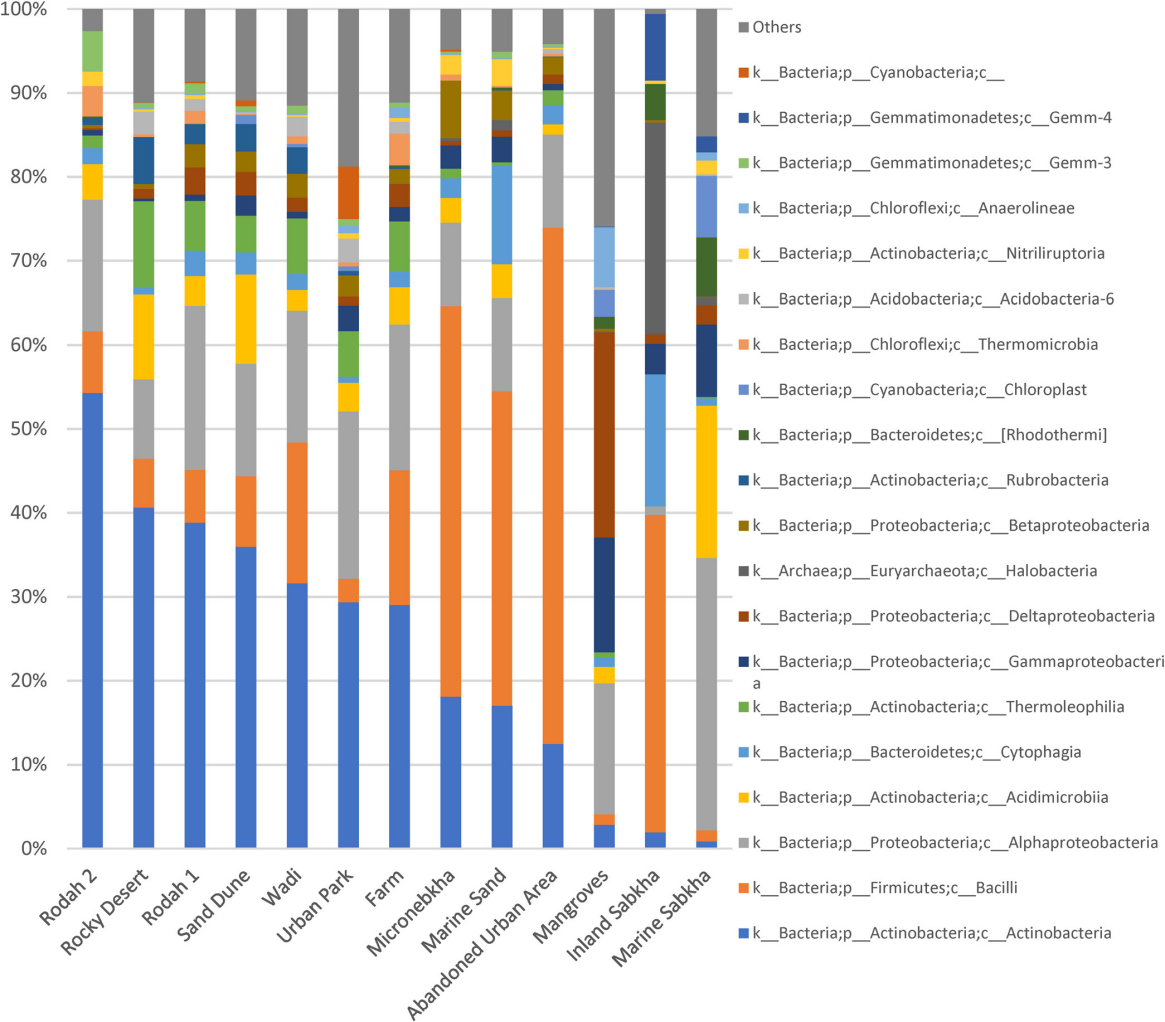
Relative abundances of the 31 most dominant bacterial and archaeal classes in each habitat using feature data to class level, ranked by *Actinobacteria* abundance.

A total of 903,258 16S rRNA gene sequences (V3-V4) were obtained for 26 soil samples, and sequences were rarified to a frequency of 8,000 reads per sample. In aggregate, the five most abundant microbial phyla accounted for 87% of all sequences, though the levels of these phyla varied dramatically between samples: *Actinobacteria* (32.3%), *Proteobacteria* (24.8%), *Firmicutes* (20.7%), *Bacteroidetes* (6.3%), and *Chloroflexi* (3.6%) (Fig. 2).

Archaeal signatures were evident in some soil samples, such as inland sabkha (24 to 25%), marine sabkha (1%), and marine sand (1.1 to 1.5%) (Fig. 2). The dominant archaeal class was *Halobacteria*, with the dominant genera being *Halorhabdus* (~10%) and *Natromonas* (~4%). Chloroplasts, more specifically *Stramenopiles*, made up 6% of marine sabkha samples and 3% of mangrove samples (Fig. 2). This subgroup includes heterotrophic protists, most likely algae. Mitochondria (*Proteobacteria; c Alphaproteobacteria;o Rickettsiales; f mitochondria*) were also noted (<0.1%). The *Archaea*, chloroplasts, and mitochondria were removed from further analysis of bacterial communities.

**FIG 3:**
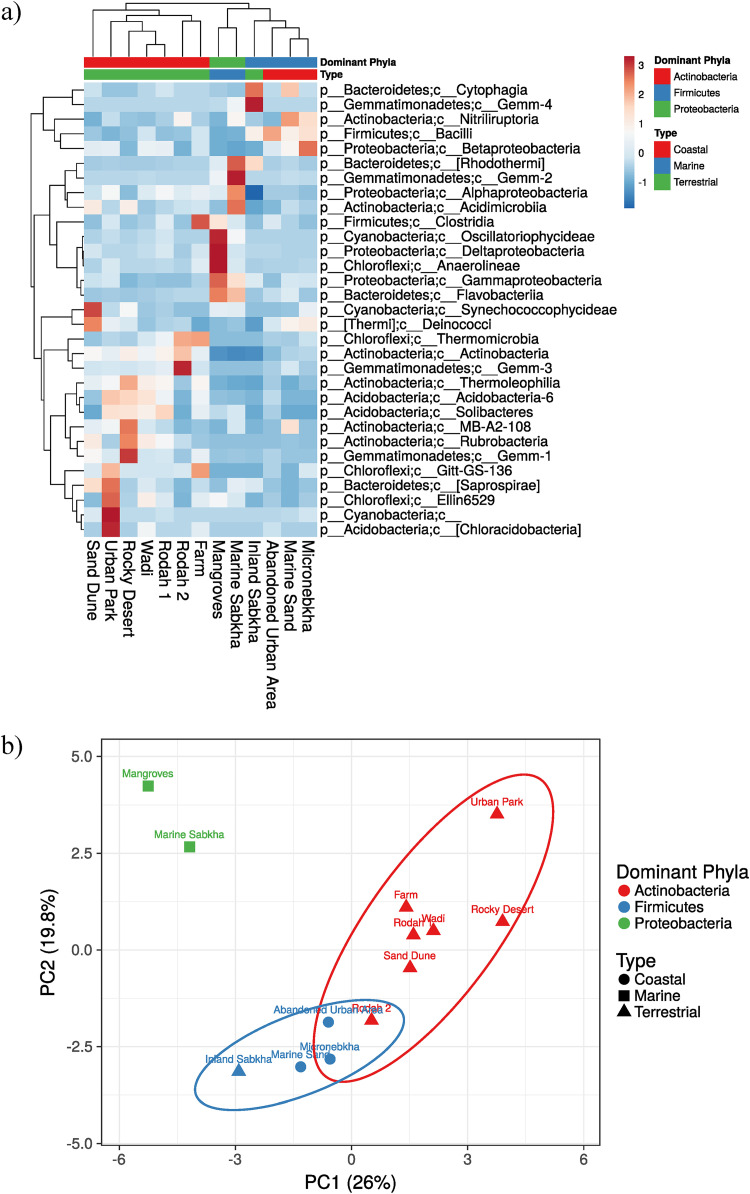
(a) Heat map showing the 31 most dominant bacterial classes in each sampled habitat. (b) PCA plot showing differences between soil sample locations and bacterial community composition (31 most dominant bacterial classes), classified by dominant bacterial phyla and type of environment, visualized with Clustvis.

Particular habitat types were dominated by specific phyla in our sampled habitats (Fig. 3a and b). Terrestrial habitats (urban park, farm, rodah, wadi, rocky desert, and sand dune) were dominated by *Actinobacteria* (39 to 62%), apart from inland sabkha, which was dominated by *Firmicutes* (Fig. 3). Coastal habitats, defined as habitats within 2 km of the coastline, such as micronebkha, marine sand, and abandoned urban area, were dominated by *Firmicutes* (37 to 62%; more specifically, *Bacilli* [Fig. 3a and b]). Marine habitats, defined as those being permanently or temporarily inundated by seawater, such as mangroves and marine sabkha, were dominated by *Proteobacteria* (43 to 56%), the former by Deltaproteobacteria and the latter by *Alphaproteobacteria* (Fig. 3a and b).

### Microbial biodiversity.

Alpha diversity, measured using feature richness [OTUs], Shannon’s entropy, and PD, varied significantly between sampled habitats using a Kruskal-Wallis test (*P* = 0.01613, *P* = 0.01648, and *P* = 0.01569, respectively) (Fig. 3 and Table 1). Feature richness and Faith’s PD varied greatly between samples (65 to 1,548 for OTUs and 6.4 to 87.9 for Faith’s PD) (Fig. 3 and Table 1). The farm samples had the highest number of OTUs (1,548) and Shannon entropy (9.3), while the inland sabkha had the lowest values for these indices (65 and 4.4, respectively) (Table 1). The mangroves had the highest Faith’s PD value (87.9), indicating high phylogenetic heterogeneity, while the inland sabkha had the lowest Faith’s PD (6.4), indicating little phylogenetic diversity (Table 1). The Spearman’s correlation between Shannon’s entropy and both OTUs (*P* = 0.0059) and Faith’s PD (*P* = 0.0040) indicates that alpha diversity is driven by both richness and phylogenetic variability in the Qatari soils we studied (Fig. S1).

**TABLE 1:** Values of alpha diversity measured by feature richness, Shannon’s entropy, and Faith’s phylogenetic diversity

Type	Shannon entropy	Faith PD	No. of observed features
Wadi	8.9	62.6	1,349
Urban park	8.8	84.0	1,434
Rodah 1	8.8	61.5	1,276
Rodah 2	7.4	37.5	706
Farm	9.3	77.2	1,548
Abandoned urban area	7.7	51.3	1,017
Marine sand	7.2	33.7	576
Mangrove	8.8	87.9	1,327
Rocky desert	7.6	38.5	717
Marine sabkha	7.3	57.4	761
Sand dune	6.9	21.8	292
Micronebkha	6.9	22.6	362
Inland sabkha	4.4	6.4	65

For beta diversity, principal-coordinate analysis (PCoA) performed on the recorded species, and calculated by Bray-Curtis dissimilarity and unweighted UniFrac, showed that the first axis explains 19.8% and 19.7% of the differences respectively, while the second axis explains 14.1 and 15.5%, respectively (Fig. S2). Bray-Curtis dissimilarity plot shows that micronebkha, marine sand, and inland sabkha samples clustered together, while mangrove and marine sabkha samples formed another cluster, and the remaining habitats clustered loosely together. This closely mirrors the alpha diversity principal-component analysis (PCA) plot (Fig. 3), apart from the abandoned urban area samples, which clustered closely with the farm samples. The unweighted UniFrac plot showed a cluster pattern similar to the Bray-Curtis dissimilarity plot apart from the sand dune samples, which clustered closely with micronebkha and marine sand samples.

### Abiotic factors of Qatari soils.

**FIG 4:**
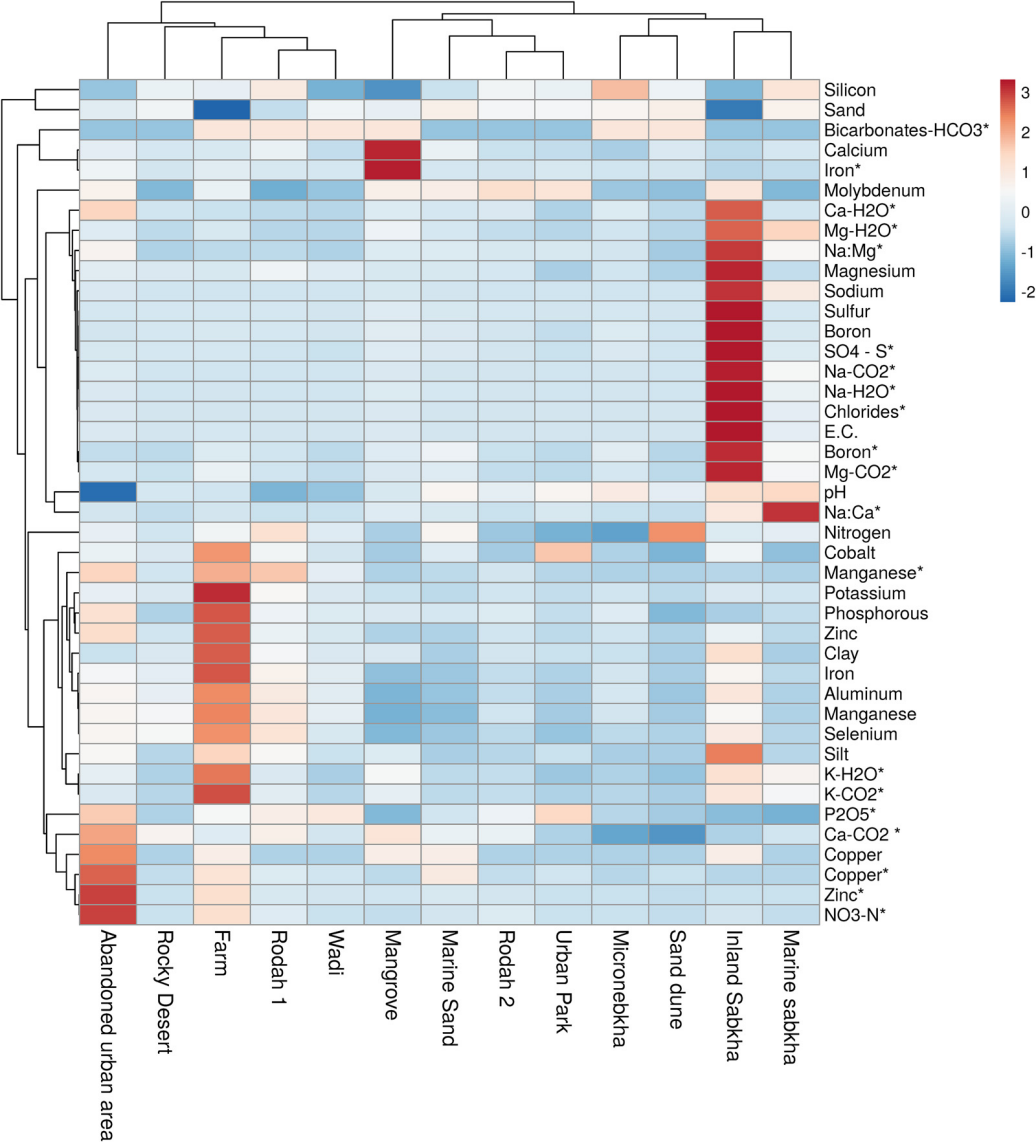
Heat map of sampled habitats and edaphic factors, visualized with Clustvis. The heat map depicts value magnitude, with red signifying high values and blue low values. E.C., EC salts; K, potassium; Na, sodium; Mg, magnesium; Ca, calcium; NO3-N, nitrates; P2O5, phosphate; SO4 – S, sulfates. Edaphic factors represent total levels except where indicated. *, available levels of the nutrient. -H2O, water-extracted, immediately available nutrient; and -CO2, carbonic acid-extracted, slowly available nutrient.

Our soil samples had an alkaline pH (7.5 to 8.2 [Table S2]), with the highest measured for marine sabkha (8.2) and the lowest measured for abandoned urban area (7.5) (Fig. 4 and Table S2). Sand was the major constituent of all the soil samples, with most samples composed of 82 to 98% sand and low levels of clay (1 to 9%) and silt (1 to 9%) (Table S2). Farm samples and inland sabkha samples were exceptions and had lower percentages of sand (56% and 64%, respectively) and higher percentages of clay (25% and 15%, respectively) and silt (19% and 21%, respectively) (Table S2). The distribution of various abiotic factors among the soil samples in this study is shown in Fig. 4.

In our samples, sodium was found to be high in sabkha samples (20,581 and 8,004 ppm for inland and marine sabkha, respectively), with inland sabkha showing additional abundances of total magnesium (20,750 parts per million [ppm]), boron (109 ppm), and sulfur (20,270 ppm) (Fig. 4 and Table S3) and available magnesium (532 and 1,370 ppm, water extracted and carbonic acid extracted, respectively), boron (12.6 ppm), chlorides (47,850 ppm), and sulfates (2,526 ppm) (Table S4). Similarly, mangrove soil samples were found to contain high levels of calcium (162,505 ppm) and available iron (32.9 ppm) (Fig. 4 and Tables S3 and S4). Sand dunes, on the other hand, had comparatively higher levels of total nitrogen (3,344 ppm) (Fig. 4 and Table S3). Farm samples showed the highest concentration for many nutrients, such as total phosphorus, potassium, zinc, iron, manganese, aluminum, selenium, and cobalt and available manganese and potassium (566, 1,614, 24, 6,236, 120, 1,978, 2.3, 1.7, 9.7, and 225 to 421 ppm, respectively) (Table S3 and Fig. 4), possibly stemming from fertilizer usage on these lands. Higher concentrations of slowly available nutrients (mean parts per million are in brackets: potassium [93.1 ppm], sodium [1,974.9 ppm], calcium [1,400.2 ppm], and magnesium [233.7 ppm]) compared to readily available nutrients (mean parts per million are in brackets: potassium [59.6 ppm], sodium [1,512 ppm], calcium [235.4 ppm], and magnesium [113.5 ppm]) were observed in our soil samples (Table S4).

Total water-soluble salts (EC salts) were highest in the sabkhas. The wadi and urban park samples had the lowest ECs (Table S4). Heavy metals like zinc and copper were dominant in disturbed areas with more anthropogenic influence (Fig. 4 and Tables S3 and S4).

### Sand, clay, and silt are key for shaping microbial diversity.

**FIG 5:**
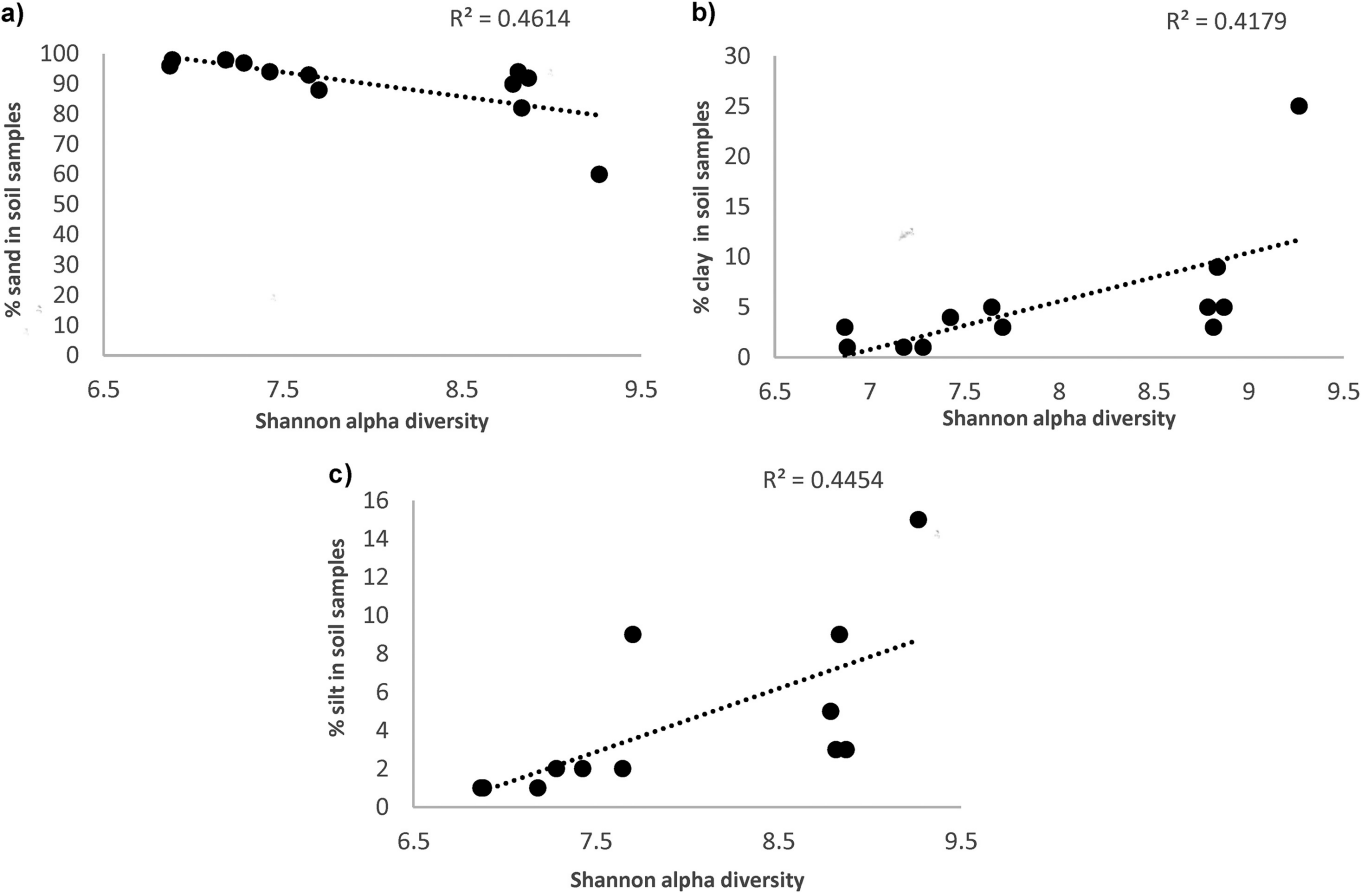
Soil edaphic factors significantly affecting Shannon diversity. Dotted line shows the trendline.

We explored the relationship between microbial alpha diversity (Shannon’s entropy) and soil texture and 40 soil chemical variables (Table S5). Sand, clay, and silt were significantly correlated with microbial diversity, using Spearman’s correlation analysis (Table 2 and Fig. 5). We found that sand had the strongest negative correlations with Shannon’s entropy (*R* = −0.84 and *P* < 0.001) (Table 2 and Fig. 5), while significant positive correlations were seen with clay (*R* = 0.79 and *P* = 0.0023) and silt (*R* = 0.89 and *P* < 0.001) (Table 2 and Fig. 5).

**TABLE 2:** Results of Spearman’s correlation analysis using Shannon entropy and soil edaphic factors as variables with Bonferroni correction^*a*^

Response variable	Correlation coefficient	*P* value
Clay	0.79	0.0023
Sand	−0.84	<0.001
Silt	0.89	<0.001

a Significance level was established at 10%, and statistically significant correlations are shown.

### Sodium correlates with specific bacterial classes.

**FIG 6:**
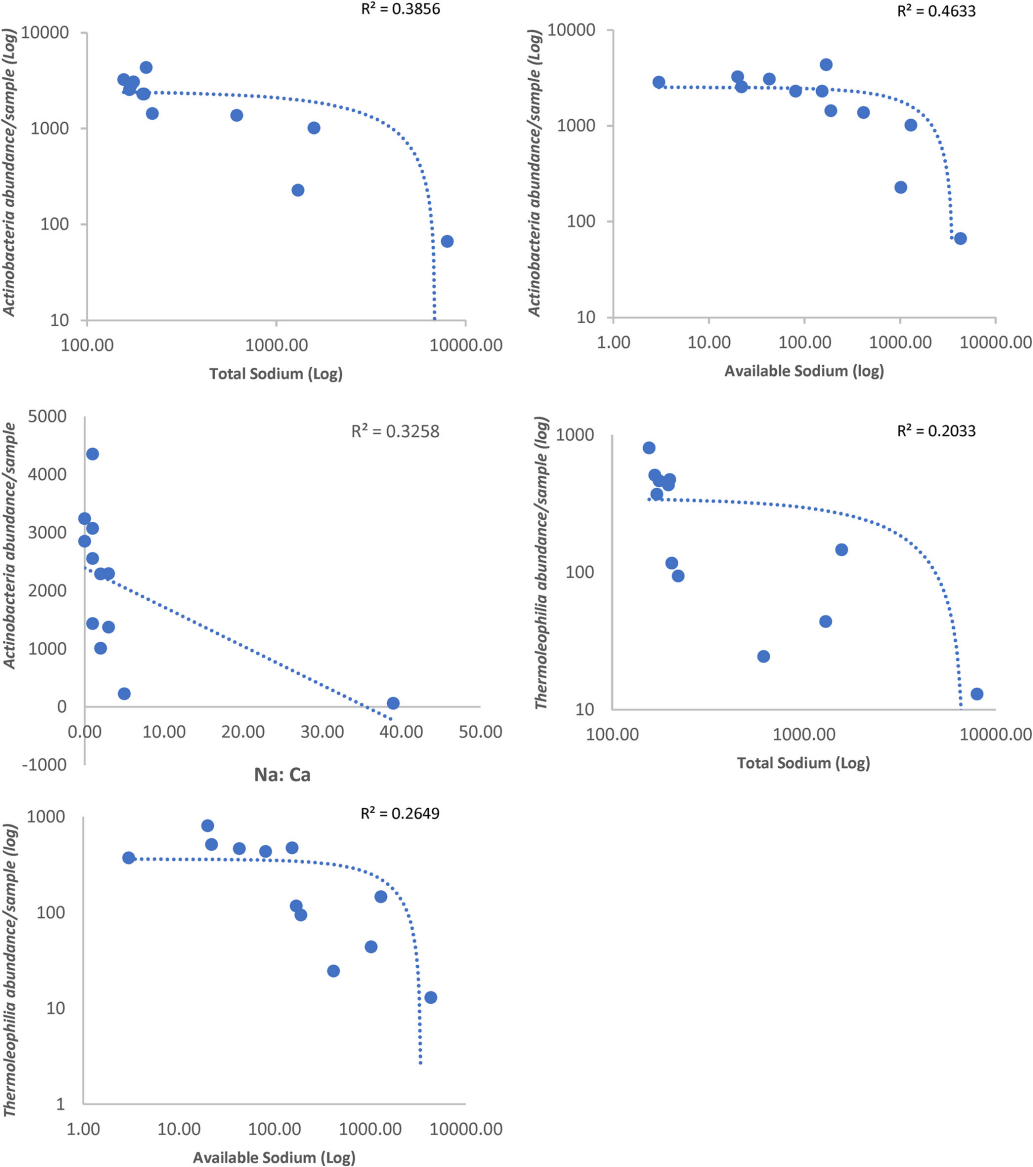
Soil edaphic factors significantly affecting specific bacterial classes. Dotted line shows the trendline.

Highly significant negative correlations were also seen at the class level between both classes *Actinobacteria* and *Thermoleophilia* (phylum *Actinobacteria*) and total sodium (*R* = −0.82 and *P* = 0.001 and *R* = −0.86 and *P* = 0.000, respectively) and slowly available sodium (*R* = −0.81 and *P* = 0.001 and *R* = −0.8 and *P* = 0.002, respectively) (Fig. 6 and Fig. S3). Additionally, the class *Actinobacteria* also showed significant negative correlation with sodium/calcium ratio (*R* = −0.81 and *P* = 0.001).

## DISCUSSION

The aim of this study was to identify the microbiota associated with different habitats present in the arid State of Qatar and to further examine the texture and chemical nature of these soils and their interrelationship.

### Microbial community composition.

The 13 natural and human-impacted habitats that were sampled revealed that the terrestrial habitats (urban park, farm, rodah, wadi, rocky desert, and sand dune) were dominated by *Actinobacteria*, apart from inland sabkha (Fig. 3). Previously, Al-Thani and Yasseen also observed a predominance of *Actinobacteria* using cultivation techniques on arid Qatari soils (10).

*Actinobacteria* have been reported to be the dominant phylum in arid environments owing to their adaptability (sporulation), wide metabolic/degradative capacity, competitive edge via antimicrobial production, and presence of multiple damage repair mechanisms (3, 34). Hence, it is no surprise that Qatari natural arid habitats such as rodah, wadi, rocky desert, and sand dune were dominated by *Actinobacteria*, similar to desert soils around the world (3). Interestingly, many novel species of *Actinobacteria* have been isolated from desert soils, suggesting that these Qatari habitats might be a productive site for bioprospecting for isolates from this phylum (3, 35, 36).

The two human-impacted terrestrial habitats (urban park and farm) revealed a similar dominance of *Actinobacteria* even though the water supply was more abundant in these two habitats. A study of organic versus conventional farming revealed that conventional farming was associated with higher relative abundance of *Actinobacteria* (37), as we observed in our farm sample. Urban parks in Qatar bear some similarity to farms in that they are intensively managed with an abundance of fertilizers, which possibly explains the abundance of *Actinobacteria* in parks (38). Urban parks in western China are also dominated by *Actinobacteria* (39), possibly due to higher microbial biomass nitrogen, which correlated with nitrogen and organic matter availability (40).

A previous study by our group on Qatari barchan dunes revealed that, on average, *Proteobacteria* dominated most of the dunes sampled. A subset of those dunes, however, were dominated by *Actinobacteria* (24). In the dune sampled for this study, *Actinobacteria* (42.9%) outnumbered *Proteobacteria* (21.2%). This difference is attributed to the fact that many of dunes sampled in the previous study (24) showed evidence of disturbance by camels, contributing to increased *Proteobacteria*. In contrast, the dune sampled for this study did not show any evidence of disturbance or proximity to a camel farm. Our dune sample also contained an abundance of *Synechococcophycidea* (*Cyanobacteria*), known to efficiently adapt to variations in salinity and light intensity (41) and *Deinococcus-Thermus*, a phylum of extremophiles.

The coastal habitats (micronebkha, marine sand, and abandoned urban area) and inland sabkha were dominated by *Firmicutes*. Members of this phylum are renowned for forming desiccation-resistant endospores, which enhance their ability to withstand extreme conditions for lengthy periods as dormant endospores (42). Others have also observed dominance of *Firmicutes* in semiarid soils in northeastern Nigeria (42) and the arid Gobi desert (43). Sabkhas are formed when intense water evaporation occurs from low-lying areas inundated with seawater or rainwater, leaving behind a dried salt-encrusted surface (12, 14, 15, 44). In our study, the most dominant archaeal class in inland sabkha was *Halobacteria*, members of which are halophilic and can tolerate extreme salinity. The majority of archaeal reads belonged to genus *Halorhabdus* (~10%), which is an extreme halophile with one of the highest salinity optima shown by microorganisms. Another dominant genus was *Natromonas* (~4%), which is an extreme haloalkaliphile. Studies show niche preferences of *Halobacteria* and *Gammaproteobacteria* in high-salinity soils, with limited diversity in these soils (45, 46), as was displayed by our inland sabkha sample (Table 1). In Saudi Arabia, the most frequently isolated bacterial species in sabkhas were Bacillus subtilis and Lactobacillus murinus (47), which were also observed in our sabkha samples. Inland sabkhas have also been associated with salt crusts containing *Cyanobacteria* of the orders *Oscillatoriales* and *Chroococcales* (48). However, only a limited presence of *Cyanobacteria* was detected in the inland sabkha soil sampled in this study, despite the presence of a salt crust on-site. Biological soil crusts are mainly comprised of cyanobacteria in the State of Qatar (13).

Members of the phylum *Gemmatimonadetes* form approximately 2% of soil bacterial communities (49). In our composite sample (all the samples combined together), a higher abundance (5.7%) was noted. This is consistent with previous reports, indicating an increased presence and adaption of these bacteria to low-moisture arid environments (49). Interestingly, the presence of bacteria such as Sphingomonas wittichii and *Novosphingobium*, known for their utilization of a wide variety of carbon sources and ability to degrade aromatic compounds (50), and *Myxococcales* such as Sorangium cellulosum, known for producing novel classes of bioactive secondary metabolites (51), was also noted overall in the Qatari soils.

The marine habitats (mangroves and marine sabkha) in this study were dominated by *Proteobacteria*. Qatar’s eastern coastline is mainly occupied by gray mangrove (Avicennia marina) stands (15, 52) and by coastal sabkhas (33). Numerous studies looking at the microbial diversity of mangroves around the world have shown predominance of *Proteobacteria* in mangroves (53–57). Like our findings, others also observed a predominance of sulfate reducers in mangrove soil, most prominently Deltaproteobacteria (53). This is attributed to the anoxic conditions typically present in mangroves as well as the low redox potential, which offers ideal conditions that select for sulfate reducers (58). In line with previous findings by Al-Thani et al. (33), we also observed a dominance by *Alphaproteobacteria* in marine sabkha in Qatar. Members of *Alphaproteobacteria*, especially purple nonsulfur bacteria, can survive sabkha conditions, as they are photoheterotrophic bacteria that have bacteriochlorophylls to capture light energy and other carotenoid pigments that protect against damaging UV radiation. These microorganisms can also be chemotrophic when surviving in the dark (59). Members of the class *Anaerolineae* (*Chloroflexi*) are regarded as core microbial populations in anaerobic digesters (60) and were relatively abundant in our mangrove samples (Fig. 2).

Rodah 2 had a relatively lower abundance of reads of the actinobacterial class *Thermoleophilia* than other habitats dominated by *Actinobacteria*. Lower *Thermoleophilia* reads were also evident in habitats dominated by *Firmicutes*, hence the overlap between rodah 2, micronebkha, and abandoned urban area in Fig. 3. *Thermoleophilia* are abundant in hot spring and soil samples, where they are reported to play an important role in biogeochemical cycling (61).

### Microbial biodiversity and abiotic factors of Qatari soils.

Similar to our findings (Table S2), Babiker (4) also found that Qatari soils are predominantly alkaline in nature and low in nutrients, which is consistent with desert soils in general. Soil edaphic factors play a greater role than plant factors in shaping the microbial diversity and composition in soil (62). Studies differ on the importance of various edaphic factors towards microbial richness, structure, and diversity, due to the fact that each soil ecosystem is a complex and dynamic environment arising from the myriad interactions between the prevalent edaphic factors and other climatic and environmental conditions, resulting in unique spatial niches supporting microbial life (63). Microbes in desert soils around the world have been shown to be influenced by salinity (45), pH and carbon (64), moisture, and organic matter (65), among other factors.

Globally, It has been shown that soils from agricultural lands, hot deserts, grasslands, and shrublands show higher alpha diversity/richness, than forests, cold deserts, and tundra biomes (66). Similarly, we also noticed varying alpha diversities within the various habitats sampled in this study. The farm sample in our study was finer textured than our average samples and had more micronutrients owing to fertilizer usage, which may have contributed to it having the highest microbial diversity among all the samples under study (Shannon entropy = 9.3).

In our study, urban park exhibited a high diversity and thus has the potential to form ecological mosaics and provide a habitat for various species within the disturbed areas of the city. Although urban park (Shannon entropy = 8.8) and farm (Shannon entropy = 9.3) were highly vegetated (~80%), as opposed to rodah 1 (~10% cover; Shannon entropy = 8.8) and wadi (~10% cover; Shannon entropy = 8.9), they all exhibited a relatively high alpha diversity (Table S1) and contained an abundance of *Rhizobiales*, a group of alphaproteobacteria associated with nitrogen fixing and plant roots. Rodah 2 displayed lower alpha diversity (Shannon entropy = 7.4) and contained several halophytic plants, consistent with its higher salinity levels (Table S3). Rodahs typically have a higher content of organic matter than other habitats in the desert (12), presumably contributing to high microbial diversity. The differences in microbial diversity between the two rodahs in this study could be due to the fact that the second rodah was more saline. Differences in nutrient compositions were also noted between the two sites, with rodah 1 having larger total amounts of most nutrients, with the notable exception of higher levels of sodium (total and available), molybdenum, EC, available chlorides, sulfates, readily available calcium and magnesium in the second rodah.

Micronebkhas have been described as water reservoirs that provide “fertile islands” for the growth of desert annuals (59). The micronebkha we sampled had lower sodium than average among studied samples, which may contribute to its potential to support plant growth. The disturbed urban area in our study had high concentrations of heavy metals like zinc and copper due to traffic emissions and urban dust, as others have observed (67) (Fig. 4; see also Table S4 in the supplemental material).

The mangrove soil in our study showed relatively high concentrations of calcium and water-soluble iron (Fig. 4 and Tables S3 and S4). Calcium levels have been reported to contribute to microbial diversity previously (68). In yet another study, exchangeable calcium correlated with microbial community at the domain and subphylum levels in soil samples from southwest China (69). Calcium controls various soil factors such as pH, preserves bicarbonate equilibrium, and is intricately intertwined with organic and inorganic carbon, which explains its role in contributing to microbial diversity (69). pH was recently shown to be an important predictor of mangrove community composition (57). Iron solubility in seawater is extremely low; however, concentrations of soluble iron tend to be high in wetlands and mangroves. Hinokidani and Nakanishi have proposed that solubility of iron in mangrove sediments is helped by the interaction of 3 factors: the presence of mangrove-derived phenolic compounds, the consumption of leaves, and the deposition of feces by crabs and snails, all of which help solubilize iron (70).

### Sand, clay, and silt are key for shaping microbial diversity.

In a previous study from Israel, it was concluded that in arid/semiarid soils, environmental factors are more vital in shaping bacterial community composition than geographic distances and spatial distribution patterns (65). Soil bacterial community structure and abundance in arid/semiarid and dry Mediterranean areas have been previously shown to be significantly correlated with clay/sand/silt composition, similar to the findings of our study (65). Others studying soil texture and microbial diversity observed relatively higher levels of bacterial richness and diversity in finer-textured soils (71, 72), which falls in line with our findings. Our data also show that clay (Spearman’s correlation coefficient = 0.79) and silt (Spearman’s correlation coefficient = 0.89) were positively correlated with bacterial diversity, while sand showed a negative correlation (Spearman’s correlation coefficient = −0.84). Soil texture affects the moisture level and EC of the soil (73). The size of soil particles can create different spaces for soil to hold the water particles (73). Sand-rich soils have larger particle size and lower surface area and hence do not hold moisture very well and have low EC (73). Silt has a medium EC value. Soils with increased clay content have higher water retention capacity and higher EC (73). Changes in moisture content greatly impact microbial diversity, especially at low moisture levels (74), which supports our results. We have observed that soils with a higher sand percentage and presumably lower moisture levels negatively impact bacterial diversity, while silt and clay-rich soils with presumably higher water holding capacity positively correlate with bacterial diversity.

Another important factor to consider that explains our results is that soil texture is intricately correlated with cation exchange capacity (CEC) range. CEC defines the soil’s ability to bind and hold nutrients with positively charged ions such as calcium (Ca^2+^) and potassium (K^+^), among others (75). CEC influences soil structure stability, nutrient availability, soil pH, and the soil’s reaction to fertilizers and other ameliorants (76). Sandy soils have low CEC (less than 3 centimol positive charge per kg of soil), while clay-rich soils have a much higher CEC (75) and can potentially bind to more nutrients and support microbial growth.

### Sodium correlates with specific bacterial classes.

In arid/semiarid soils, actinobacterial abundance has been shown to remain unaffected by differences in the sand and clay contents of the soil (65), as seen in the soil samples from this study. Salinity may pose a problem in arid and semiarid soils (77). Sensitive microorganisms are killed by high salt content due to osmotic stress, whereas some bacteria can adapt and become salt tolerant like halophiles (77). In agricultural soils, the relative abundance of *Actinobacteria* was shown to decrease with salinity (78). Another study from China shows that soil salinity is negatively related to the relative abundances of both *Actinobacteria* and *Thermoleophilia* (79). These studies support our results, which also show that *Actinobacteria* and *Thermoleophilia* are negatively correlated with total sodium and slowly available sodium levels in Qatar soils. High sodium concentrations are also known to cause soil dispersion, clay platelet and aggregate swelling that hardens soil and blocks water infiltration (80), and thus likely affect microbial growth of salt intolerant microbes.

### Study limitations and conclusions.

We realize that our study has a limited sample size, which affects the statistical power of the analysis and may pose a limitation to our study. In this study, we collected 2 soil samples per site (within a 2-m radius of each other) from 13 distinct habitats in Qatar and used this to base the overall soil microbiome composition of various habitats present in Qatar. While it does not give absolute resolution, given the relatively small size of the State of Qatar, with a total area of 11,610 km^2^ (81), the limited samples are an attempt to analyze the similarities and variances present in the different habitats in Qatar. Furthermore, this study has the advantage of using 16S rRNA sequencing to detect the overall bacterial composition (culturable and nonculturable) of multiple habitats, in contrast to previous studies from Qatar studying only cultured bacteria or using sequencing technology on soil from specific habitats. Another limitation to this study is that because of the use of the 16S rRNA gene for sequencing, only the bacterial community present in Qatari soils could be characterized, precluding the identification of associated fungi. Furthermore, because of the wide seasonal and diurnal temperature variations in Qatar, a single-time-point study as in this case may miss out on the full dynamic range of microbial consortia present in these soils.

In conclusion, we characterized the different soil bacterial communities present in Qatar using 16S rRNA gene sequencing. Our results show that Qatari soils are home to diverse microbes, which vary from one habitat to the other. Different habitats in Qatar showed varied microbial profiles, with most terrestrial habitats, coastal areas, and marine water inundated habitats showing predominance of *Actinobacteria*, *Firmicutes*, and *Proteobacteria*, respectively. Soil texture was found to correlate with microbial diversity in these arid lands. The abundance of *Actinobacteria* warrants further research into these soils, particularly as potential sources of novel biologically active small compounds.

## MATERIALS AND METHODS

### Soil sample collection.

Soil was collected over a period of 3 days in May 2017. A total of 26 samples were collected from 13 habitats (described in the introduction) in the State of Qatar (Fig. 1). Two biological replicate samples were collected (within a 2-m radius) at each habitat using a sterile 10-cm-deep standard steel bulb planter (Fiskars Brands, Inc., Finland). The corer was disinfected with 90% ethanol, washed with distilled water, and dried after each habitat was sampled. Samples were kept on ice in the field and during transport to the laboratory (~1 to 2 h). Once in the laboratory, samples were aliquoted and stored at −80°C until further processing. These habitats were selected to capture the maximum landform diversity in samples based on ecological knowledge of the country and ease of access. At each sample point, the GPS coordinates, time, air and soil temperatures were recorded in addition to notes on vegetation cover, anthropogenic influence, and any other unique features.

### Analysis of soil chemical properties.

Soil samples were submitted to Texas Plant & Soil (TPS) Lab, Edinburg, TX, for detailed salinity, pH, electrical conductivity and principal-micronutrient analysis. Available nutrients were extracted by the CO_2_ extraction method (Plant Natural) based on the work of Daubeny (82, 83). In this method, potassium (K), sodium (Na), calcium (Ca), and magnesium (Mg) were extracted using carbonic acid to extract slowly available nutrients as well as with water to identify water-soluble/readily available nutrients. Soil texture was determined by measuring the settling rates of primary particles in an aqueous solution using the Bouyoucos hydrometer method (84). Total nutrient levels were extracted using the Aqua Regia total extraction method (85–88).

### DNA extraction and 16S rRNA sequencing.

All 26 samples were processed and initially analyzed using ZymoBIOMICS Service—targeted metagenomic sequencing (Zymo Research, Irvine, CA). The ZymoBIOMICS DNA miniprep kit (Zymo Research) was used for DNA extraction from the soil according to the manufacturer’s instructions. Bacterial 16S primers, custom-designed by Zymo Research, were used to amplify the V3-V4 region of the 16S rRNA gene. The Quick-16S NGS library preparation kit (Zymo Research) was used for library preparation (89). The PCR products were quantified with quantitative PCR (qPCR) fluorescence readings and pooled based on equal molarity. The final pooled library was cleaned up with Select-a-Size DNA Clean & Concentrator (Zymo Research) and then quantified with TapeStation and Qubit. The library was sequenced on Illumina MiSeq with a version 3 reagent kit (600 cycles).

### Data analysis.

Bioinformatics analysis was carried out using the ZymoBIOMICS Service—targeted metagenomic sequencing (Zymo Research, Irvine, CA) pipeline. Unique amplicon sequences were inferred from raw reads using the Dada2 pipeline version 1.16 (90). Chimeric sequences were also removed with the Dada2 pipeline. Taxonomy assignment was performed using Qiita version .0.1.0-dev (91). Approximately ~903,000 sequences were rarified to a frequency of 8,000 reads per sample. A 16S closed-reference biological observation metric (BIOM) table was created in Qiita, whereby every sequence was matched to the Greengenes database version 3_8-97 by a 97% sequence identity threshold and given a taxonomic assignment. Archaea and chloroplasts were filtered out and removed from further bacterial analysis. Alpha diversity was measured using feature richness, Shannon diversity index, and Faith’s phylogenetic diversity (PD) with Qiita. Beta diversity was quantified directly from variations in the 10 most abundant phyla using complete linkage cluster and PCA plots in Clustvis version 2.0 (92). Additionally, both Bray-Curtis dissimilarity and unweighted UniFrac analyses were performed and visualized as PCoA plots in Qiita. In all cases, biological replicates grouped closely with each other; therefore, postsequencing, data from the two biological replicates for each site were pooled and treated as one to give a more comprehensive microbiome profile for each site.

### Statistical analysis.

Spearman’s correlations were conducted to assess the effects of (i) soil texture and pH, (ii) total nutrient concentration, and (iii) available nutrient concentrations of various chemical factors on bacterial diversity and the abundances of specific bacterial taxa (top 5% at class level in abundance). The significance level was established at 10%. Due to multiple testing, an adjustment to the *P* value was conducted using the Bonferroni method. Given the extreme values for many of the chemical measurements for the inland sabkha habitat, this sample was excluded from the correlation tests in order to discern trends that were relevant to the rest of the samples.

### Data availability.

We have deposited all 16S rRNA gene sequence data associated with this article in the NCBI Sequence Read Archive under accession number PRJNA613915.
