# Supplementary material for: Soil Properties Correlate with Microbial Community Structure in Qatari Arid Soils
Source: Microbiol Spectr. 2023 Feb 27;11(2):e03462-22. doi: 10.1128/spectrum.03462-22 (PMC10100838; doi:10.1128/spectrum.03462-22)
Supplement: Supplemental file 1 — Supplemental material. Download spectrum.03462-22-s0001.pdf, PDF file, 2.8 MB [file spectrum.03462-22-s0001.pdf]

Supplementary Data:

Table 1: Table listing description of the sampled habitats. na; not available.

Figure 1: The correlation between Shannon's Entropy and a) OTU (p=0.0059) and b) Faith's PD (p=0.0040). OTU values were multiplied by a factor of 0.05 and Faiths PD by a factor of 0.1 to unify alpha diversity values.

Figure 2: The different Qatar habitats harbor diverse microbes and are varied from each other. Beta diversity plots (PC1 vs PC2 vs PC3) showing differences between habitats using the matrix of paired wise distance between habitats calculated by a) Brey Curtis dissimilarity and b) unweighted unifrac. Each dot on the figure represents the whole microbial composition profile of a habitat. Beta diversity (the comparison of bacterial communities based on their composition) provides a measure of the distance or dissimilarity between each soil sample pair.

Table 2: Texture and chemical analysis of the soil samples.

Table 3: Total nutrients present in the soil samples. N: Nitrogen; P: Phosphorous; K: Potassium; Na: Sodium; Ca: Calcium; Mg: Magnesium; Zn: Zinc; Fe: Iron; Mn: Manganese; Cu: Copper; B: Boron; Al: Aluminum; S: Sulphur; Si: Silicon; Mo: Molybdenum; Co: Cobalt; Se: Selenium. Units of measure: parts per million.

Table 4: Water extracted readily available and carbonic acid extracted slowly available nutrients present in the soil samples. Fe: Iron; Mn: Manganese; Cu: Copper; B: Boron; Cl: Chlorides; EC: E.C. Salts (mmhos/cm); NO3 -N: Nitrates; P2O5: Phosphate; K: Potassium; Na: Sodium; Ca: Calcium; Mg: Magnesium; HCO3: Bicarbonates; SO4 – S: Sulphates Zn: Zinc. Units of measure: NO3 -N & P2O5: lbs/ac; others: parts per million.

Table 5. Results of Spearman's correlation analysis using Shannon entropy and soil edaphic factors as variables. Significance level was established at 10% and statistically significant correlations are highlighted.

Figure 3. Heatmap of Spearman's correlation coefficients between bacterial community species at the class level and soil edaphic factors, visualized with Clustvis. Edaphic factors represent total levels except where indicated. * Represents available levels of these nutrients. -H2O indicates water extracted, immediately available, and -C02-indicates carbonic acid extracted slowly available nutrients.

Table 1: Table listing description of the sampled habitats na; not available.

No.	Habitat Name	Habitat Location	Northing	Easting	Temperature Air (°C)	Soil (°C)	Additional information
1	Dunes	Al Kharrara	25.08500 N	51.38494 E	30.5	31.5	Devoid of vegetation.
2	Rocky desert	Al Kharrara	25.08525 N	51.38507 E	33	34	Rocky desert interspersed with sandy loam. 5% vegetation cover. Dominant plants include *Zygophyllum qatarense* and *Cornulaca aucheri.*
3	Marine sabkha	Mesaieed Sabkha	24.84338 N	51.50583 E	37	36	Devoid of vegetation. Crust and soil black due to microbial anaerobic respiration from the existing water table, white salt crust present on the sample site.
4	Micronebkhas	Mesaieed	24.86379 N	51.50966 E	39	43	Lizard foot prints on site. Micronebkhas vegetated with *Seidlitzia rosmarinus.*
5	Rodah 1	South of Al Kharrara	24.98635 N	51.07345 E	42	48	10% vegetation cover. Number of mammal burrows present. Sampled sites were under *Lycium shawii* shrub.
6	Mangroves	Simaisma beach	25.57707 N	51.48813 E	28	26	40% vegetation cover consisting of mangrove *Avicennia marina*. Beach was rich in plants including *Salsola vermiculata*, crab & seaweed detritus. Soil was dark and muddy due to anaerobic respiration from the inundation of the tide.
7	Rodah 2	Dukhan	25.43520 N	51.00730 E	38	41	30% vegetation cover consisting of *Lycium shawii* , *Acacia ehrenbergiana*, and a number of grasses and halophytes such as *Zygophyllum qatarense,* and *Z. simplex*.
8	Marine sand	Dukhan beach	25.42078 N	50.75534 E	35	38	Beach had anthropogenic influence including trash, charcoal, and BBQ remains. Devoid of vegetation. Sand mixed with shells.
9	Inland sabkha	Dukhan	25.41129 N	50.87405 E	42	49	Salt crust, devoid of vegetation and muddy.
10	Wadi	Dukhan highway	25.40386 N	51.02857 E	na	na	Terrain composed of Rocky desert with sandy loam. Heavy presence of burrows. 10 % vegetation cover, dominated by *Lycium shawii.*

11	Abandoned Urban area	West Bay Lagoon	25.36086 N	51.50826 E	33	31	Vegetation cover low with presence of weed species and trash.
12	Farm	Al Jumailiya	25.56245 N	51.15638 E	34	35	Sampled site were Chili peppers and Mulukhiyah fields.
13	Urban Park	Oxygen Park	25.31120 N	51.44510 E	39	36	Sample taken from grassy regions. 80% vegetation cover.

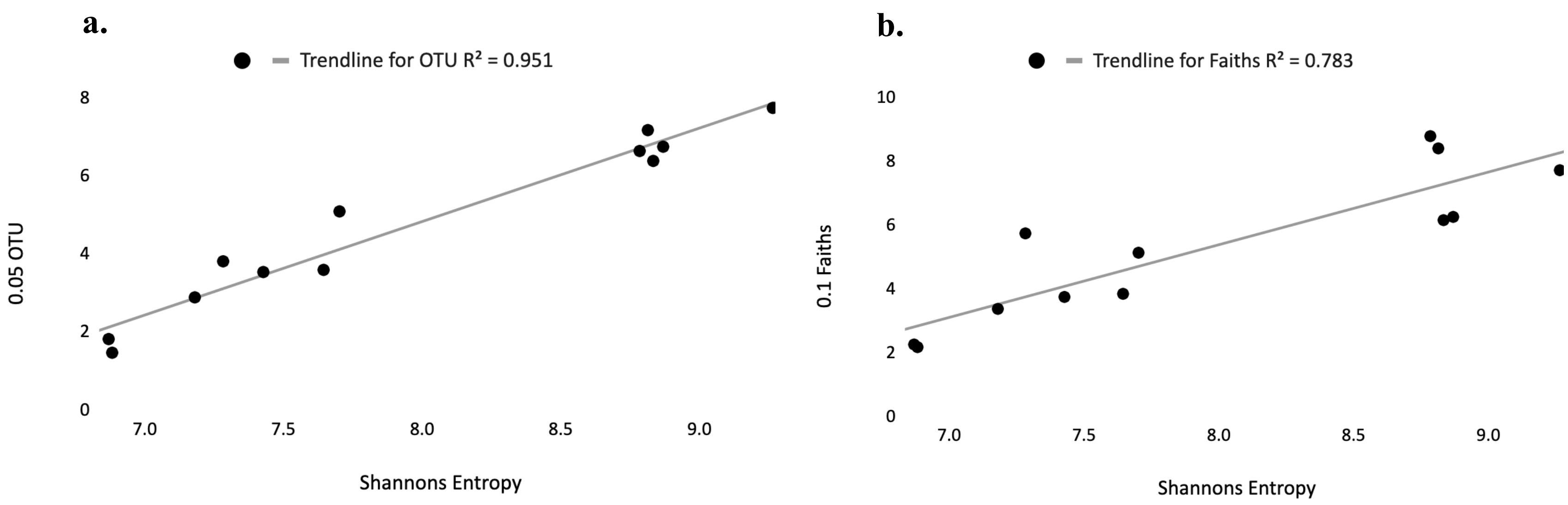

Figure 1: The correlation between Shannon's Entropy and a) OTU (p=0.0059) and b) Faith's PD (p=0.0040). OTU values were multiplied by a factor of 0.05 and Faiths by a factor of 0.1 to unify alpha diversity values.

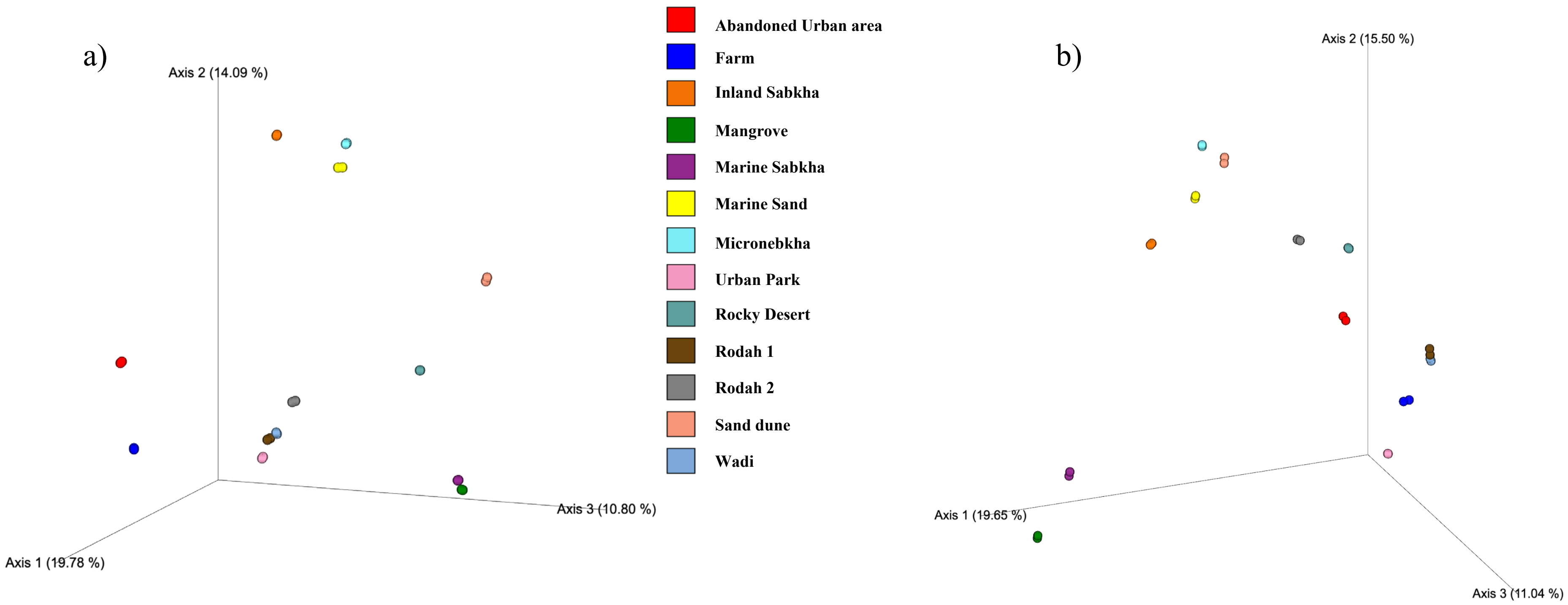

Figure 2: The different Qatar habitats harbor diverse microbes and are varied from each other. Beta diversity plots (PC1 vs PC2 vs PC3) showing differences between habitats using the matrix of paired wise distance between habitats calculated by a) Brey Curtis dissimilarity and b) unweighted unifrac. Each dot on the figure represents the whole microbial composition profile of a habitat . Beta diversity (the comparison of bacterial communities based on their composition) provides a measure of the distance or dissimilarity between each soil sample pair.

Table 2: Texture and chemical analysis of the soil samples.

Sample	% Sand	% Clay	% Silt	pH
Marine sabkha	97.00	1.00	2.00	8.19
Micronebkha	96.00	3.00	1.00	8.08
Rocky Desert	93.00	5.00	2.00	7.83
Rodah 1	82.00	9.00	9.00	7.66
Sand dune	98.00	1.00	1.00	7.90
Wadi	92.00	5.00	3.00	7.71
Inland Sabkha	64.00	15.00	21.00	8.16
Marine Sand	98.00	1.00	1.00	8.02
Rodah 2	94.00	4.00	2.00	7.91
Mangrove	90.00	5.00	5.00	7.85
Urban Park	94.00	3.00	3.00	8.02
Farm	56.00	25.00	19.00	7.81
Abandoned Urban area	88.00	3.00	9.00	7.45

Table 3: Total nutrients present in the soil samples. N: Nitrogen; P: Phosphorous; K: Potassium; Na: Sodium; Ca: Calcium; Mg: Magnesium; Zn: Zinc; Fe: Iron; Mn: Manganese; Cu: Copper; B: Boron; Al: Aluminum; S: Sulphur; Si: Silicon; Mo: Molybdenum; Co: Cobalt; Se: Selenium. Units of measure: parts per million.

Sample	N	P	K	Na	Ca	Mg	Zn	Fe	Mn	Cu	B
Marine sabkha	2000.00	108.00	164.00	8004.00	47261.00	3198.00	3.00	1144.00	24.00	1.00	13.00
Micronebkha	1115.00	170.00	175.00	221.00	33842.00	3778.00	4.00	1474.00	34.00	1.00	15.00
Rocky Desert	1716.00	79.00	250.00	156.00	48629.00	4797.00	4.00	2137.00	59.00	1.00	10.00
Rodah 1	2707.00	213.00	559.00	176.00	63734.00	7207.00	8.00	3086.00	79.00	1.00	10.00
Sand dune	3344.00	26.00	91.00	171.00	52430.00	2344.00	2.00	872.00	20.00	1.00	7.00
Wadi	1929.00	157.00	253.00	167.00	41249.00	5361.00	5.00	1990.00	49.00	1.00	9.00
Inland Sabkha	1876.00	73.00	251.00	20581.00	39408.00	20750.00	8.00	2995.00	63.00	2.00	109.00
Marine Sand	2318.00	140.00	104.00	617.00	64097.00	4483.00	2.00	774.00	14.00	2.00	14.00
Rodah 2	1433.00	160.00	163.00	205.00	42664.00	4142.00	4.00	1240.00	33.00	1.00	9.00
Mangrove	1504.00	151.00	137.00	1301.00	162505.00	4445.00	2.00	602.00	9.00	2.00	18.00
Urban Park	1256.00	106.00	114.00	197.00	40627.00	2139.00	3.00	1013.00	21.00	1.00	5.00
Farm	2194.00	566.00	1614.00	200.00	49043.00	4661.00	24.00	6236.00	120.00	2.00	12.00
Abandoned urban	2053.00	346.00	404.00	1577.00	59991.00	5550.00	15.00	2707.00	64.00	3.00	9.00

Sample	Al	S	Si	Mo	Co	Se
Marine sabkha	553.00	603.00	22807.00	0.04	0.11	0.70
Micronebkha	733.00	643.00	28269.00	0.09	0.24	0.72
Rocky Desert	961.00	631.00	15604.00	0.04	0.48	1.32
Rodah 1	1315.00	615.00	21528.00	0.01	0.77	1.70
Sand dune	463.00	522.00	16128.00	0.06	0.03	0.66
Wadi	875.00	429.00	4193.00	0.08	0.40	0.93
Inland Sabkha	1382.00	20270.00	5150.00	0.48	0.75	1.55
Marine Sand	441.00	962.00	10080.00	0.43	0.55	0.57
Rodah 2	642.00	574.00	16995.00	0.53	0.20	0.74
Mangrove	326.00	2055.00	698.00	0.42	0.19	0.44
Urban Park	515.00	393.00	15374.00	0.49	1.39	0.55
Farm	1978.00	861.00	15063.00	0.31	1.69	2.33
Abandoned urban	1156.00	772.00	6354.00	0.41	0.68	1.41

Table 4: Water extracted readily available and carbonic acid extracted slowly available nutrients present in the soil samples. Fe: Iron; Mn: Manganese; Cu: Copper; B: Boron; Cl: Chlorides; EC: E.C. Salts (mmhos/cm); NO3 -N: Nitrates; P2O5: Phosphate; K: Potassium; Na: Sodium; Ca: Calcium; Mg: Magnesium; HCO3: Bicarbonates; SO4 – S: Sulphates Zn: Zinc. Units of measure: NO3 -N & P2O5: lbs/ac; others: parts per million

Sample	Fe	Mn	Cu	B	Cl	E.C.	NO3 -N	P2O5	K-H2O	K-CO2	Na-H2O
Marine sabkha	1.19	0.29	0.02	3.66	5350.00	17.33	4.00	4.00	105.00	145.00	2319.00
Micronebkha	2.37	0.40	0.02	2.12	339.00	2.38	12.00	21.00	16.00	19.00	99.00
Rocky Desert	2.53	1.34	0.09	0.22	99.00	0.98	13.00	18.00	15.00	23.00	11.00
Rodah 1	3.81	8.84	0.30	0.64	63.00	0.73	57.00	61.00	48.00	95.00	37.00
Sand dune	2.87	0.37	0.12	0.03	25.00	0.40	6.00	15.00	2.00	6.00	3.00
Wadi	2.99	3.04	0.15	0.20	18.00	0.35	9.00	66.00	11.00	23.00	13.00
Inland Sabkha	0.15	0.51	0.04	12.60	47850.00	153.60	21.00	9.00	141.00	215.00	14665.00
Marine Sand	3.02	0.82	0.91	2.93	398.00	2.26	22.00	27.00	22.00	30.00	379.00
Rodah 2	2.67	1.54	0.08	0.46	335.00	1.87	49.00	46.00	27.00	35.00	111.00
Mangrove	32.92	0.41	0.06	1.81	2060.00	8.16	7.00	8.00	92.00	105.00	999.00
Urban Park	3.60	0.50	0.14	0.10	45.00	0.35	11.00	79.00	5.00	18.00	36.00
Farm	5.42	9.71	1.03	1.70	419.00	2.53	213.00	52.00	225.00	421.00	118.00
Abandoned urban	8.31	8.21	1.85	0.32	2415.00	8.71	411.00	83.00	66.00	75.00	866.00

Sample	Na-CO2	Ca -H2O	Ca -CO2	Mg-H2O	Mg-CO2	HCO3	SO4 - S	Na: Ca	Na: Mg	Zn
Marine sabkha	4312.00	110.00	1284.00	350.00	403.00	2.00	238.00	39.00	12.00	0.14
Micronebkha	189.00	208.00	1028.00	54.00	124.00	3.00	204.00	1.00	4.00	0.11
Rocky Desert	20.00	114.00	1590.00	25.00	67.00	2.00	108.00	0.00	1.00	0.20
Rodah 1	43.00	65.00	1619.00	21.00	84.00	3.00	43.00	1.00	2.00	0.66
Sand dune	3.00	66.00	969.00	19.00	30.00	3.00	25.00	0.00	0.00	0.09
Wadi	22.00	38.00	1306.00	17.00	55.00	3.00	3.00	1.00	1.00	0.40
Inland Sabkha	17944.00	1083.00	1221.00	532.00	1370.00	2.00	2526.00	17.00	34.00	0.17
Marine Sand	416.00	156.00	1461.00	68.00	207.00	2.00	179.00	3.00	6.00	0.64
Rodah 2	169.00	181.00	1463.00	34.00	59.00	2.00	86.00	1.00	5.00	0.34
Mangrove	1023.00	207.00	1705.00	159.00	172.00	3.00	261.00	5.00	6.00	0.29
Urban Park	81.00	32.00	1211.00	18.00	32.00	2.00	2.00	3.00	5.00	0.20
Farm	153.00	100.00	1383.00	78.00	308.00	3.00	108.00	2.00	2.00	3.62
Abandoned urban	1299.00	700.00	1963.00	100.00	127.00	2.00	164.00	2.00	13.00	6.96

Table 5. Results of Spearman's correlation analysis using Shannon entropy and soil edaphic factors as variables. Significance level was established at 10% and statistically significant correlations are highlighted.

Test 1: Texture and pH

% Sand	R	-0.839
	P-value	<0.001
% Clay	R	0.786
	P-value	0.0023
% Silt	R	0.886
	P-value	<0.001
pH	R	-0.658
	P-value	0.02

Test 2: Total nutrients

Nitrogen	R	0.077
	P-value	0.812
Phosphorous	R	0.42
	P-value	0.175
Potassium	R	0.608
	P-value	0.036
Sodium	R	-0.287
	P-value	0.366
Calcium	R	0.091
	P-value	0.779
Magnesium	R	0.51
	P-value	0.09
Zinc	R	0.579
	P-value	0.048
Iron	R	0.503
	P-value	0.095
Manganese	R	0.503
	P-value	0.095
Copper	R	0.193
	P-value	0.547
Boron	R	-0.205
	P-value	0.524
Aluminum	R	0.503
	P-value	0.095
Sulfur	R	-0.056
	P-value	0.863
Silicon	R	-0.497
	P-value	0.101
Molybdenum	R	-0.014
	P-value	0.966
Cobalt	R	0.629
	P-value	0.028
Selenium	R	0.441
	P-value	0.152

Test 3: Available nutrients

Zinc	R	0.634
	P-value	0.027
Iron	R	0.622
	P-value	0.031
Manganese	R	0.727
	P-value	0.007
Copper	R	0.515
	P-value	0.087
Boron	R	-0.252
	P-value	0.43
Chlorides	R	-0.147
	P-value	0.649
E.C.	R	-0.203
	P-value	0.527
NO3-N	R	0.329
	P-value	0.297
P2O5	R	0.559
	P-value	0.059
K-H2O	R	0.273
	P-value	0.391
K-CO2	R	0.417
	P-value	0.178
Na-H2O	R	-0.028
	P-value	0.931
Na-CO2	R	-0.196
	P-value	0.542
Ca -H2O	R	-0.476
	P-value	0.118
Ca -CO2	R	0.378
	P-value	0.226
Mg-H2O	R	-0.189
	P-value	0.557
Mg-CO2	R	0.007
	P-value	0.983
HCO3	R	0.241
	P-value	0.45
SO4 - S	R	-0.385
	P-value	0.216
Na:Ca	R	0.136
	P-value	0.673
Na:Mg	R	-0.141
	P-value	0.662

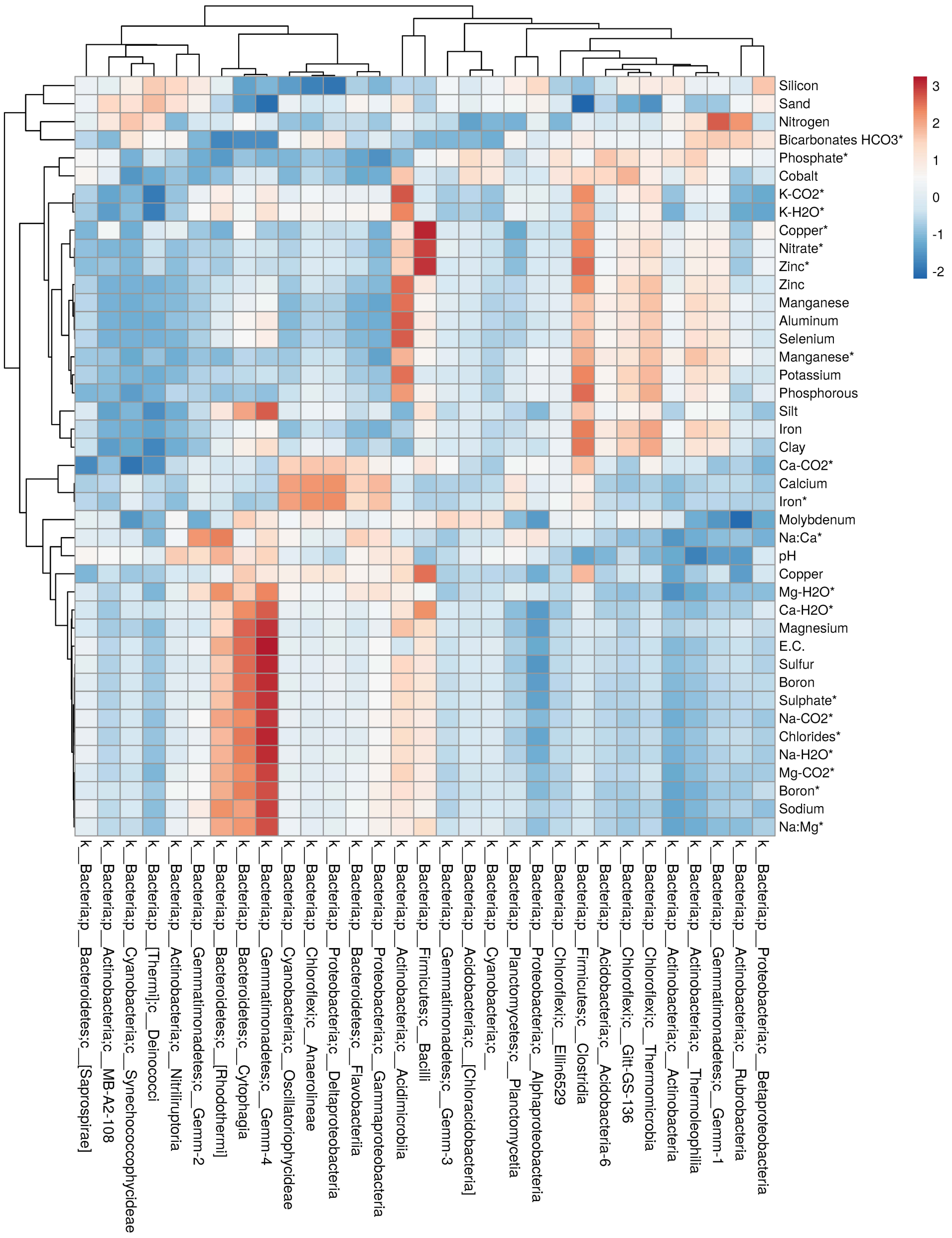

Figure 3. Heatmap of Spearman's correlation coefficients between bacterial community species at the class level and soil edaphic factors, visualized with Clustvis. Edaphic factors represent total levels except where indicated. * Represents available levels of these nutrients. -H2O indicates water extracted, immediately available, and -C02- indicates carbonic acid extracted slowly available nutrients.
